# Evaluating Cefoperazone-Induced Gut Metabolic Functional Changes in MR1-Deficient Mice

**DOI:** 10.3390/metabo12050380

**Published:** 2022-04-22

**Authors:** Jinchun Sun, Zhijun Cao, Ashley D. Smith, Paul E. Carlson Jr, Michael Coryell, Huizhong Chen, Richard D. Beger

**Affiliations:** 1 Division of Systems Biology, National Center for Toxicological Research, United States Food and Drug Administration, Jefferson, AR 72079, USA; zhijcao@gmail.com (Z.C.); richard.beger@fda.hhs.gov (R.D.B.); 2 Laboratory of Mucosal Pathogens and Cellular Immunology, Division of Bacterial, Parasitic, and Allergenic Products, Office of Vaccines Research and Review, Center for Biologics Evaluation and Research, United States Food and Drug Administration, Silver Spring, MD 20993, USA; ashdawnsmith@gmail.com (A.D.S.); paul.carlson@fda.hhs.gov (P.E.C.J.); michael.coryell@fda.hhs.gov (M.C.); 3 Division of Microbiology, National Center for Toxicological Research, United States Food and Drug Administration, Jefferson, AR 72079, USA; huizhong.chen@fda.hhs.gov

**Keywords:** metabolomics, microbiome, metabolic functioning biomarkers, carbohydrates

## Abstract

Mucosal-associated invariant T cells are activated following the recognition of bacterial antigens presented by the major histocompatibility complex class I-related molecule (MR1). Previous metagenomics data showed that MR1^−/−^ knock-out (KO) mice had distinct microbiota and displayed a resistance to *Clostridioides difficile* (CDI) colonization vs. wild-type (WT) mice. In the present study, LC/MS-based untargeted metabolomics are applied to evaluate the changes in metabolic activities, in accordance with the changes in gut microbiota caused by cefoperazone (Cef) treatment. Adult C57Bl/6J WT and MR1^−/−^ KO mice were given sterile drinking water or spiked with 0.5 mg/mL Cef ad libitum for five days. Fecal pellets were collected daily, and both small intestinal and cecal contents were harvested at sacrifice. The PLS-DA score plots of the metabolomic data indicate that the microbiota is relatively less disturbed by Cef treatment in KO mice, which is consistent with the metagenomics data. The most noticeable differences in the metabolome of KO and WT mice were the increases in carbohydrates in the WT mice, but not in the KO mice. Metabolic functional biomarkers were identified through the correlation analysis of gamma-aminobutyric acid (GABA) and riboflavin. These detected metabolic functional biomarkers could provide information complementary to metagenomics data.

## 1. Introduction

Vertebrates host hundreds of trillions of intestinal microbes, which develop symbiotic relationships with the host [1]. The gut microbiota plays important roles in salvaging energy and absorbable nutrients, as well as in supporting the host immune system development and maintenance, which are important to the host’s physiology and pathology status [2,3]. The diverse intestinal microbiota is critical in sustaining a homeostatic equilibrium state of microbial composition and function through host–microbial and microbial–microbial interactions [4,5]. Dysbiosis, a disruption of the microbial homeostatic equilibrium, may lead to many diseases, such as inflammatory bowel disease (IBD), colon cancer, obesity, and asthma [6,7]. Gut microbial balance can be influenced by alterations in diet [8,9], antibiotic use [10,11], and other environmental factors [12]. Among these, antibiotic-induced disturbances in the intestinal microbial communities and functions are unable to fully recover, even months after the discontinuation of dosing [13]. In some cases, the recovered gut ecosystem contains bacterial species that are different from those existing prior to the antibiotic intervention, but possess similar functions [5]. Therefore, it is important to discover functional metabolic biomarkers, which can characterize “what the microbes do” in addition to determining “what the microbes are”.

Cefoperazone (Cef) is a broad-spectrum antibiotic used to treat skin infections, intra-abdominal infections, respiratory infections, and urinary tract infections. Previous studies [14,15,16] have shown that Cef administration caused significant microbiota changes in the small and large intestines; the small intestine contains more diverse microbiota than the large intestine. The Cef-altered intestinal microbiota were reduced in diversity and abundance resulting in altered metabolic activities. These were reflected by the intestinal metabolome changes, specifically, decreases in secondary bile acids, glucose, free fatty acids, and dipeptides, whereas increases in primary bile acids and sugar alcohols were also reported [15]. Our previous study showed similar results, where increases in primary bile acids and decreases in secondary bile acids were observed in mice after Cef administration [17]. Another study also showed that decreases in many host-gut microbiota co-metabolites (indole- and phenyl-containing metabolites, amino acids, vitamins, nucleotides, and bile acids) were observed in the urine from mice treated with penicillin (an antibiotic used to treat Gram-positive bacterial infections) [18].

As a follow-up study [17], LC/MS-based untargeted metabolomics was employed to investigate metabolic activity changes in the small intestinal content, cecal content, and stool from MR1^−/−^ (KO) and wild-type (WT) mice after Cef administration. MR1, the major histocompatibility complex class I-related molecule, is able to bind to bacteria-synthesized riboflavin metabolites (antigens), which can be presented to mucosal-associated invariant T cells (MAIT) for activation [19,20] to defend against some microbial activity and infection [21]. Previous studies showed that the fecal microbiota of KO mice is more diverse than that of the WT mice after Cef treatments [16,22]. In this work, our goal is to discover metabolic biomarkers as microbiota functional indicators to reflect gut microbiota changes after Cef administration to both KO and WT mice.

## 2. Results

### 2.1. Gut Metabolome Alteration by Cef Treatments

LC/MS-based untargeted metabolomics was conducted to measure metabolic alterations in small intestinal content, cecal content, and stool to observe whether metabolic alterations reflected distinct microbiota populations at different sites in the gut. The identified metabolites, mean intensities, and significant changes are summarized in Table S1. Since bile acid data has already been previously published [17], this article focuses on other metabolite changes. In total, 90, 204, and 256 annotated metabolites were detected in stool, small intestinal content, and cecal content, respectively (Table S1).

**Figure 1:**
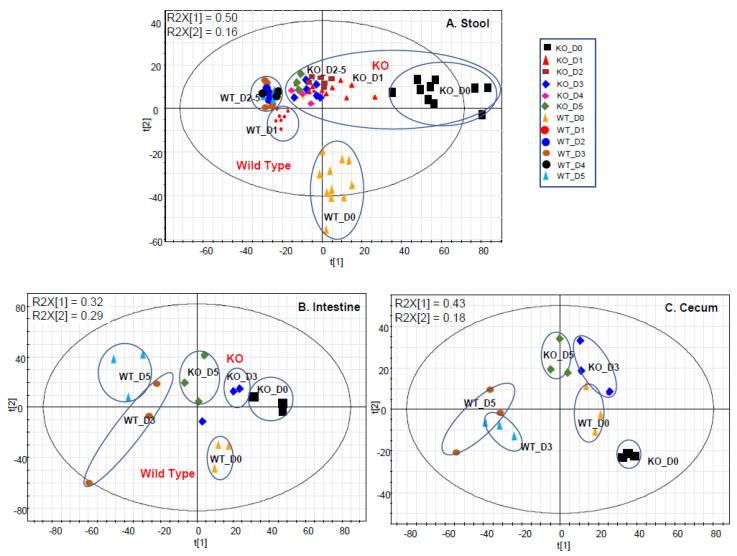
The PLS-DA score plots for stool (**A**) at D0 (control), D1, D2, D3, D4, and D5; and the small intestinal content (**B**) and cecal content (**C**) at D0 (control), D3, and D5. KO indicates the knock-out mice and WT indicates wild type.

The score plots of the PLS-DA analysis of the metabolome data show group separations (Figure 1). In general, (i) the KO mice were clearly separated from the WT mice; and (ii) the distance of the dosed groups away from the D0 group in KO mice was closer than the distance in the WT mice for all the sample types. From a visual inspection, the distance of the dosed groups to the D0 group is the closest in the small intestinal contents (Figure 1A) from KO mice, compared to those group distances for the stool (Figure 1B) or cecal content (Figure 1C) samples. The distance indicates the extent of the metabolome alterations induced by Cef treatments. The greater the distance, the more significant the disturbance. Less metabolic alterations in the KO mice were consistent with microbiome changes that the microbiome was more diverse, which led to less metabolic alterations in the KO mice after Cef dosing.

**Figure 2:**
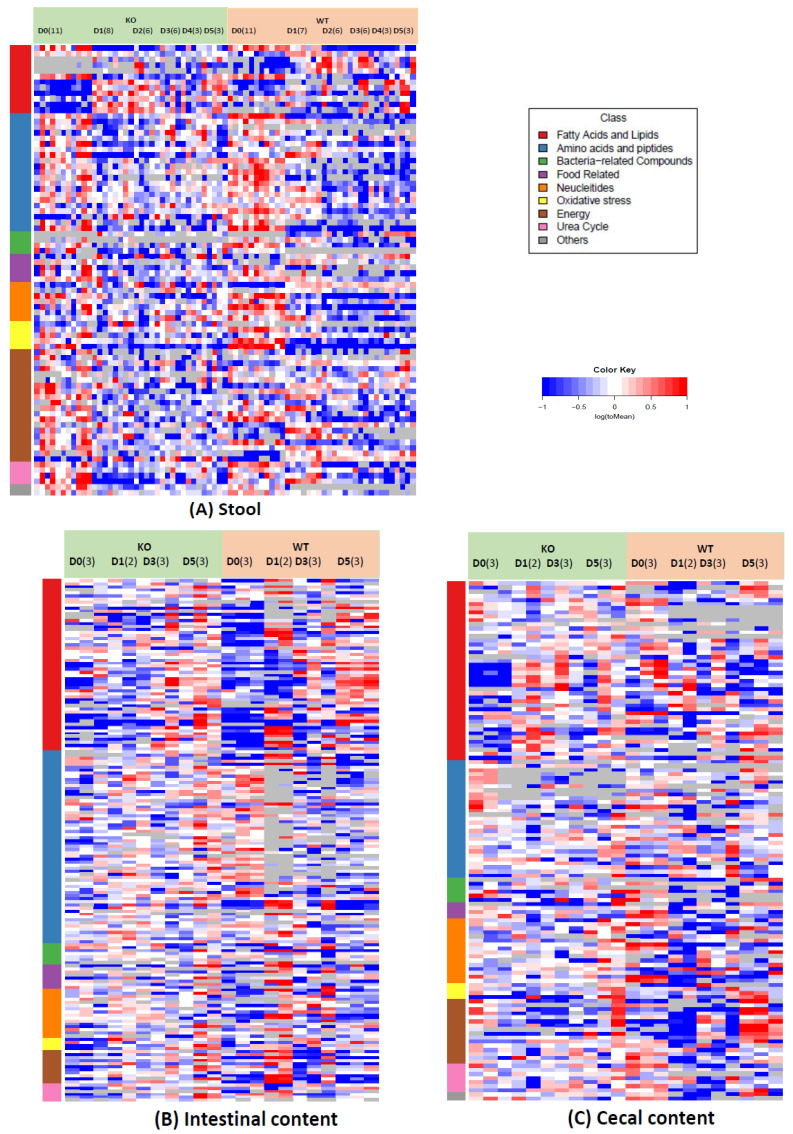
Untargeted metabolomics of the gut metabolome. Heat maps of log_e_ transformed normalized intensity of individual metabolite for stool samples (**A**), small intestinal content (**B**), and cecal content (**C**). A metabolite normalized intensity is the intensity divided by the average for each metabolite in all of the animals of each group. The metabolites are grouped by the Kyoto Encyclopedia of Genes and Genomes pathways as lipids, amino acids, bacteria-related compounds, food-related compounds, nucleotides, oxidative stress, energy, and urea cycle. Each row represents the log_e_ (normalized intensity) of a metabolite displayed in the order listed in Table S1.

Based on the Kyoto Encyclopedia of Genes and Genomes (KEGG) pathways and the metabolite origination, the detected metabolites were classed as lipids, amino acids (AAs) and peptides, bacteria-related compounds, food-related compounds, nucleotides, oxidative stress-related compounds, carbohydrates, and urea cycle compounds (Figure 2, Table S1). In order to attain a global view of the changes of all of the detected metabolites by Cef treatments, the heat maps of the metabolites from all the sample types are displayed in Figure 2. Figure 2 shows that the metabolites exhibited the same dose-related trends in both KO and WT mice, but to a lesser extent in KO mice (Figure 2). Figure 2 also displays that stool samples had clearly more alterations in the metabolomes, compared to either small intestinal metabolome changes or cecal metabolome changes. In the stool samples, AAs, peptides, short-chain fatty acids (SCFAs) and bacteria-related metabolites drastically decreased, while some carbohydrates clearly increased in the WT mice dosed with Cef (Figure 2A). In the small intestinal contents, the largest increases were observed in the medium-chain fatty acids, lysoPCs, and PCs in the dosed animals. However, those same compounds increased in the KO mice to a lesser extent (Figure 2B). In the cecal contents, decreases in the SCFA and bacteria-related metabolites, and increases in some carbohydrates, were observed in both the KO and WT mice dosed with Cef (Figure 2C).

**Figure 3:**
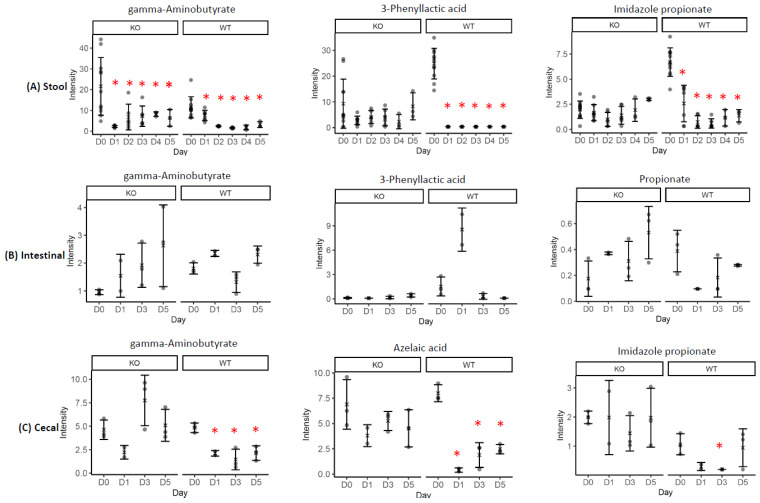
Box plots of the intensity of the selected bacteria-associated metabolites in the stool (**A**), small intestinal (**B**), and cecal contents (**C**) from both the KO and WT mice. Whereas D0 = prior to the Cef treatment, D1, 2, 3, 4, and 5 means 1, 2, 3, 4, or 5 days after the Cef treatments. *: significant changes at *p* < 0.05. Error bars denote the standard deviations (SDs).

Figure 3 displays the box plots of the selected SCFAs and bacteria-related metabolites metabolites including gamma-aminobutyrate (GABA), *3*-phenyllactic acid, and imidazole propionate intensity levels present in stool samples (Figure 3A); GABA, phenyllactic acid and propionate in small intestinal contents (Figure 3B); and GABA, azelaic acid, and imidazole propionate in cecal contents (Figure 3C).

### 2.2. Correlations of the GABA or Riboflavin to Gut Metabolites

The correlation analysis of the stool data (Table S2) shows that GABA had strong correlations with metabolites (i) involved in the GABA biosynthesis pathway, including glutamate (Pearson’s correlation coefficient *r* = 0.92), acetylserine (*r* = 0.84), *N*-acetylaspartate (*r* = 0.69), alanine (*r* = 0.62), pyruvate (*r* = 0.53), and aspartate (*r* = 0.46)); (ii) vitamin biosynthesis pathways, including riboflavin (*r* = 0.67), nicotinate (*r* = 0.77), and pantothenic acid (*r* = 0.79); and (iii) carbohydrates, including 1,5-anhydroglucitol, glucuronate, citraconate/glutaconate, and quinate with 0.4 < *r* < 0.54. In stool, GABA has significant inverse correlations with lipids and carbohydrates, including ribose, xylonate/arabinonate, and quinate (Table S2). The correlation analyses of the small intestinal content and cecal content (Table S2) show similar correlation results, in that GABA maintained strong correlations with the metabolites involved in GABA biosynthesis and vitamin biosynthesis pathways. The small intestinal metabolome correlation analysis also shows that GABA has a significant correlation with propionic acid (*r* = 0.51), a SCFA produced by bacteria (Table S2). The cecal metabolome correlation analysis shows that GABA has a significant correlation with many other bacteria-related metabolites, including 2-oxindole-3-acetic acid, imidazole propionate, and *N*-(2-furoyl)glycine with 0.5 < *r* < 0.65. Similar to the stool correlation data, the cecal GABA has significant strong inverse correlations with carbohydrates, including gulonate and xylonate/arabinonate (Table S2).

The correlation analysis of the stool data (Table S3) shows that riboflavin has strong inverse correlations with carbohydrates, including quinate, citraconate/glutaconate, arabinonate, ribose, and myo-inositol/glucose with a correlation coefficient 0.4 < *r* < 0.5. Riboflavin also had strong correlations with metabolites (i) involved in the oxidative stress pathway, including glutamate, SAMe, and methionine; (ii) vitamin biosynthesis pathways, including nicotinate and pantothenic acid; and (iii) bacteria-related metabolites, including GABA, *N*-(2-furoyl)glycine and phenyllactic acid with 0.4 < *r* < 0.67. Similar results were observed in the small intestinal data, where riboflavin had strong correlations with other vitamins (nicotinate and pyridoxal), and with bacteria-related metabolites, including phenyllactic acid, 3-hydroxypropanoate, and homoveratric acid sulfate (Table S3). In the cecal correlation data, riboflavin had significantly inverse correlations with carbohydrates (ribose, xylonate/arabinonate, and gulonate) with *r* ~ 0.6. The cecal riboflavin also had strong correlations with pantothenic acid and other bacteria-relates metabolites (*N*-(2-furoyl)glycine and 2-oxindole-3-acetic acid) (Table S3).

### 2.3. Pathway Impact Analysis

**Figure 4:**
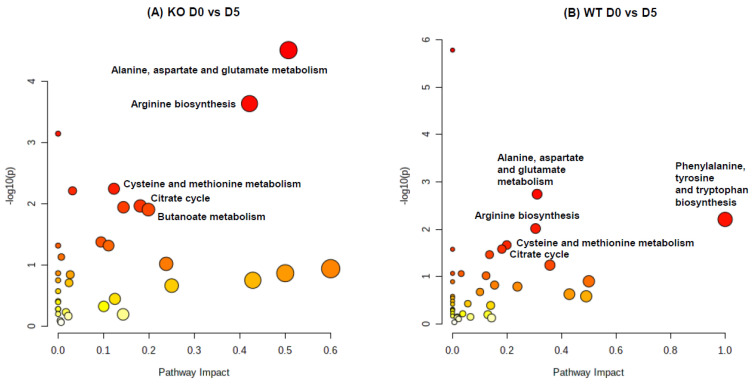
Metabolome view of the pathway impact analysis obtained from significantly changed metabolites in stool from Cef treatments in KO (**A**) and differential metabolites in WT mice (**B**). This analysis was conducted using MetaboAnalyst (www.metaboanalyst.ca, accessed on 1 January 2022). The color and size of each circle is based on *p*-values (yellow: higher *p*-values, and red: lower *p*-values) and pathway impact values (the larger the circle, the higher the impact score) calculated from the topological analysis, respectively. The pathways were considered as significantly impacted if *p* < 0.05, the impact is 0.1, and the number of metabolite hits in the pathway > 1.

The metabolome overview of the pathway impact of significantly changed metabolites in stool (KO D0 vs. D5 and WT D0 vs. D5) using MetaboAnalyst (www.metaboanalyst.ca, accessed on 1 January 2022) is displayed in Figure 4. The pathway analysis of significantly altered metabolites shows perturbations in alanine, aspartate and glutamate metabolism (involved in GABA biosynthesis), arginine biosynthesis, cysteine and methionine metabolism, and citrate cycle, with common influences on both the KO and WT mice after Cef treatments.

## 3. Discussion

Antibiotics can disrupt the gut microbial community by reducing the diversity and abundance of intestinal microbiota, which often leads to pathogen infections, including *Clostridioides difficile* infection (CDI). We previously demonstrated that MR1^−/−^ KO mice had a distinct microbiota composition compared to WT mice, both prior to and after treatment with the broad-spectrum antibiotic, cefoperazone [16]. Prior to the antibiotic treatment, the microbiota of the KO mice was composed of 40% of *Porphyromonadaceae* (vs. 32% in WT), 15% of *Lachnospiraceae*, and ~22% of *Lactobacillaceae* (vs. 15% in WT), with less unclassified *Clostridiales* and *Erysipelotrichaceae* than the microbiota in the WT mice. After the Cef treatments, the microbiota of the KO mice had a similar composition to that prior to treatment, while WT mice exhibited dramatic microbiome alterations, in that ~95% of the population was composed of *Enterococcaceae* and *Bacteroidaceae*. MR1 binding with the antigen is needed for MAIT cell (a subset of T cells in the immune system) activation. As such, it is no surprise to find out that the gut microbiota community (directly related to the host immune system) of KO mice was distinct from the WT mice. KO mice were resistant to CDI, even following an antibiotic treatment. CDI resistance was transferrable when microbes of KO mice were transferred to WT mice via fecal microbiota transplantation (FMT) [16]. Analyzing the microbiome, based on 16S or metagenomic sequencing, provides microbial composition information, but is unable to provide information regarding the transcriptional activity of the genes and is unable to differentiate between live or dead microbes, or directly investigate functional metabolites. Here, LC/MS-based metabolomics were conducted to discover the metabolic functional biomarkers of the gut microbiota, which could provide complementary information to metagenomics data. This was based on the hypothesis that MR1^−/−^ KO and Cef treatments cause the gut microbiota community to alter, and the altered gut microbiota further induce metabolome changes. Therefore, the changed metabolites could reflect the gut microbiota bioactivity changes.

From a global view, the close cluster of all KO groups for all the sample types in the PLS-DA score plots (Figure 1) of the metabolome data indicated that the microbiota was relatively less disturbed by Cef treatments in the KO vs. WT mice. This is consistent with the microbiome data, showing that stool microbial diversity was less changed by Cef treatments in KO mice [16]. Furthermore, the cluster of KO groups in the PLS-DA score plot (Figure 1) of the metabolome data from small intestinal contents is tighter than those from stool or cecal contents. This suggests that Cef caused less microbiota disturbance in the small intestine compared to that in the large intestine, which has also been confirmed by others [14].

**Figure 5:**
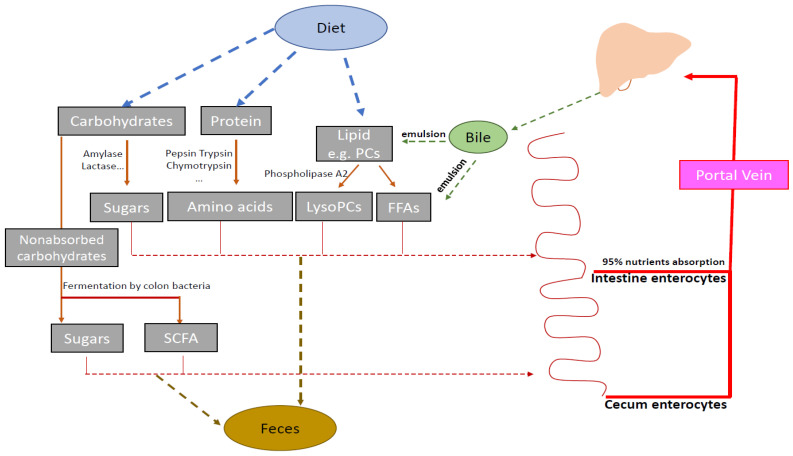
Nutrition digestion and absorption in the gut.

The most noticeable metabolite changes in the small intestinal contents consisted of increases in lipids (Figure 2B and Table S1) after Cef dosing. In the small intestine, PCs from diet dissolved in the emulsification of bile, and were hydrolyzed by the pancreatic secreted phospholipase A2 to lysoPCs and FFAs (Figure 5). The digested lysoPCs, FFAs, acyl carnitines, or other lipids emulsified with bile are required for absorption by the intestine. Significant decreases in the total bile acids were observed in both the WT and KO mice (to a lesser extent, decreases in the KO mice) after dosing [17], which could be the cause of the slow absorption of digested lipids and result in lipid accumulation in the small intestinal content. Since little-to-no accumulation was observed in either the cecal contents or stool samples after Cef dosing (Table S1), the overall absorption of lipids was not ultimately influenced by Cef dosing.

The most striking class of the changed metabolites was that of carbohydrates, which significantly increased in Cef-dosed WT mice, with less dramatic changes in the stool or cecal content samples of Cef-dosed KO mice (Table S1). Weaker inverse correlations of GABA or riboflavin to carbohydrates were observed in the Cef-dosed KO mice vs. WT mice (Tables S2 and S3), which might be correlated to gut microbiota being less affected by Cef dosing in KO [16] mice. After Cef treatments, the stool levels of carbohydrates, including xylonate/arabinonate, glutaconate, ribose, and myo-inositol/glucose, which also had significant correlations with GABA or riboflavin, were higher in the WT mice vs. KO mice (Tables S2 and S3). The greatest fold changes were ~7 folds in xylonate/arabinonate (WT(D5)/KO(D5) = 45.64/6.70), while there were ~2.7, 1.6, and 1.8 folds in glutaconate, ribose, and myo-Inositol/glucose, respectively (Table S1). It must be noted that xylonate and arabinonate coeluted, as well as myo-inositol and glucose, which made it impossible to separately quantify these carbohydrates using the current LC method.

Xylonate/arabinonate (sugar acids) is known for being an energy source for some bacteria containing xylose/arabinonate dehydrogenase, including *G. oxydans*, *pseudomonas putida*, and *E. coli* [23,24]. Increases in these carbohydrates—which could be the essential energy source for pathogens, such as *C.* *difficile*—might confer CDI susceptibility in WT mice. Indeed, Theriot et al. [15] discovered *that* nutrient media supplemented with glucose, fructose, mannitol, or sorbitol promote *C.* *difficile* growth in vitro. The same study also revealed similar metabolic findings, namely, increases in carbohydrates, including those observed in this study, were observed in the WT mice after a 10-day dosing period. Most recently, it was reported that *E. coli* added in the synthetic murine bacterial community containing 12 species, conferred resistance to *Salmonella* infection by competing for galactitol, a carbohydrate [25]. Strowig and colleagues [26] found that fecal samples from children with a resistance to *Klebsiella* *pneumoniae* colonization contained high levels of *K. oxytoca.* A further in vivo study revealed that the resistance was due to *K. oxytoca* outcompeting for *β*-glucoside sugars, which are essential for *K. pneumoniae*. Therefore, the lower levels of carbohydrates (including xylonate/arabinonate, glutaconate, ribose, and myo-Inositol/glucose) might be a plausible reason for the observed resistance to CDI in KO mice. This requires further investigation.

GABA is primarily synthesized from glutamate, and also known as a microbial metabolite produced by many Gram-negative and Gram-positive bacteria, such as *Lactobacillus* and *Bifidobacterium* [27]. Riboflavin, a water-soluble vitamin, can be obtained from food, and can also be biosynthesized by bacteria, such as *Bifidobacterium*, in the large intestine. Therefore, the metabolic function or function-related biomarkers could be identified through the correlations of GABA or riboflavin to the gut metabolome. In the stool correlation analysis of GABA (Table S2), potential metabolic functional biomarkers included glutamate, acetylserine, *N*-acetylaspartate, alanine, pyruvate, and aspartate, which are directly or indirectly involved in GABA biosynthesis pathways, as shown in Figure 4. Potential metabolic functional biomarkers in the stool with a strong correlation to GABA also included vitamins (pantothenic acid, riboflavin, and nicotinate) and *N*-(2-furoyl)glycine, a bacteria-related metabolite. Strong correlations of GABA or riboflavin to *N*-(2-furoyl)glycine suggest that the common gut microbiota involved in the three metabolite biosynthesis pathways were depleted by Cef dosing in both the WT and KO mice. The stool correlation analysis of riboflavin (Table S3) discovered potential metabolic functional biomarkers, including methionine, glutamate, and SAMe, which are all involved in oxidative stress pathways noted in Figure 4. The strong correlation of the oxidative stress-related metabolites and vitamins (including pantothenic acid and nicotinate) to riboflavin is understandable, since riboflavin coenzymes (FAD and FMN) and nicotinate coenzymes (NAD and NADP) are involved in redox reactions. Furthermore, antibiotic-induced imbalanced gut microbiota can cause higher levels of reactive oxygen species (ROS) in the intestinal environment [28]. Therefore, the levels of vitamins and oxidative stress-related metabolites could reflect the redox state changes caused by Cef-induced gut microbiota alterations.

## 4. Materials and Methods

### 4.1. Chemicals

Optima LC/MS grade acetonitrile and water were purchased from Thermo Fisher Scientific Inc. (Pittsburgh, PA, USA). Metabolite standards were obtained from Sigma-Aldrich (St. Louis, MO, USA). Cef was obtained from the MP Biomedicals (Santa Ana, CA, USA).

### 4.2. Animal Care and Treatment

All animal experiments were previously described [16,17]. The animal experiments were conducted at the Center for Biologics Evaluation and Research, United States Food and Drug Administration (FDA), and were reviewed and approved by the FDA Institutional Animal Care and Use Committee (Approval # 2015-08 ASP). Adult C57Bl/6J or MR1^−/−^ C57Bl/6J mice were administered water or sterile water containing 0.5 mg/mL Cef ad libitum for 1, 2, 3, 4, or 5 days; water was refreshed every other day. Fecal pellets were collected daily throughout the duration of the experiment (*n* = 11, 8, 6, 6, 3, and 3 biological replicates at D0, D1, D2, D3, D4, and D5, respectively, for both the KO and WT groups). In the present study, D0 is considered as the control, while D1, 2, 3, 4, and 5 are samples collected after 1-, 2-, 3-, 4-, and 5-day treatments, respectively. After the collection, the fecal samples were immediately stored at −80 °C for sequencing and metabolomics analysis. A total of 2 to 3 mice from the KO or WT groups were sacrificed at D0, 1, 3, and 5. At sacrifice, the small intestinal content and cecum content were collected (*n* = 3, 2, 3, and 3 at D0, D1, D3, and D5, respectively). After collection, the small intestinal and cecal contents were immediately snap frozen, then stored at −80 °C, until metabolomics analysis was performed.

### 4.3. Quality Control in Metabolomics

A standard mixture quality control (QC) sample, comprised of 40 common chemicals for LC/MS open profiling, was evaluated [29] to monitor the analytical equipment variability. Pooled sample QC data were acquired from every 10 sample runs to monitor the analytical equipment variability and for data filtering.

### 4.4. Open Metabolic Profiling by UPLC/QTof-MS

The metabolites were extracted, as previously described, by mixing the small intestinal and cecal contents (~250 mg) with extraction solvent (1:1 MeOH: water) at a ratio of 1:5 (*w*/*v*), or a ratio of 1:10 (*w*/*v*) for the stool pellets [17]. After sonication and centrifugation, the supernatant was then transferred to autosampler vials for metabolomics analysis.

The metabolites were separated using a Waters Acquity Ultra Performance Liquid Chromatography (UPLC) system (Waters, Milford, MA, USA) equipped with a Waters bridged ethyl hybrid (BEH) C8 column with a dimension of 2.1 mm × 10 cm and a 1.7 µm particle size. Mass spectrometric data were collected with a Waters QTof Premier mass spectrometer (Waters, Milford, MA, USA) operated in positive and negative ionization electrospray modes. The details of the UPLC method and the parameter settings for QTof-MS were previously described [17].

Raw UPLC/MS data were analyzed using Micromass MarkerLynx XS Application vers. 4.1 (Waters, Milford, MA, USA) with extended statistical tools. The same parameter settings for the peak extraction from the raw data were used, as previously reported [30,31]. The aligned data from the MarkerLynx analysis for the QTof-MS data were filtered using pooled QC samples based on the following criteria: (i) ions with %RSD less than 30% in the pooled QC samples were included, and (ii) ions present in ≥70% of QC samples were included. The resulting dataset was analyzed by supervised partial least squares discriminant analysis (PLS-DA) with Statistica vers. 12.1 (Statsoft, Tulsa, OK, USA). The identification of compounds was based on the combined information of accurate mass measurements and fragmentation mass spectra. Annotation confidence levels for each metabolite were provided, based on the Metabolomics Standards Initiative (MSI) confidence scheme [32], and the annotation confidence levels are noted in Table S1. Peak annotation was first performed by matching the accurate mass measurement (<6 ppm), retention time (<0.02 min), and fragmentation spectra to an in-house library (level 1). Other metabolites were identified by matching *m/z* (<6 ppm) (level 3) and fragmentation spectra to an opensource online database [33,34] (www.hmdb.ca, accessed on 1 January 2022) (level 2).

### 4.5. Statistics

In the metabolome data analyses, the missing values were replaced with half of the minimum values of all the samples for each metabolite. A fixed effect linear regression model was used for the statistical analysis of the log_e_ transformed intensity of each metabolite from the small intestine and cecum samples. Since multiple samples from the same animal were collected at different time points, a mixed-effects linear regression model was used for the statistical analysis of the log_e_ transformed intensity of each metabolite from the stool samples. The effect of the Cef treatment was estimated by contrasting data at D1, D2, D3, D4, or D5 with data at D0. The effect of KO was estimated by contrasting data from the KO mice with data from the WT mice. The Benjamini and Hochberg method was used to calculate the false discovery rate (FDR) across the analytes [35]. Data visualization, correlation analysis, and statistical analysis were performed using R software [36].

## 5. Conclusions

Cef treatments altered the gut microbiota in terms of diversity and abundance in both the KO and WT mice, but to a lesser extent in KO mice. However, the KO mice showed a resistance to CDI, even following antibiotic treatments. The LC/MS-based untargeted metabolomics study displayed that striking increases in certain carbohydrates (essential for the proliferation of *C.* *difficile*) were present in the WT mice, but not in KO mice. The correlation analysis of GABA or riboflavin identified metabolites involved in the GABA pathway (glutamate, acetylserine, *N*-acetylaspartate, alanine, pyruvate, and aspartate); the oxidative stress pathway (methionine, glutamate, and SAMe); vitamins (pantothenic acid and nicotinate); and *N*-(2-furoyl)glycine. These detected metabolic function biomarkers could provide information complementary to the metagenomics data and could serve as indicators of the functional status of the gut microbiota (formed by the repertoire of expressed genes and by proteins and metabolites). Furthermore, the levels of carbohydrates in the stool may be good indicators of a susceptibility to CDI, which needs further investigation.

## Data Availability

The data presented in this study are available in the article and Supplementary Materials.

## Supplementary Materials

The following supporting information can be downloaded at: https://www.mdpi.com/article/10.3390/metabo12050380/s1; Table S1: Detected metabolites extracted from fecal samples, small intestinal contents, and cecal contents collected from the KO or WT mice; Table S2: List of metabolites from fecal samples, in small intestinal and cecal contents, which has a significant correlation with GABA; and Table S3: List of metabolites from fecal samples, in small intestinal and cecal contents, which has a significant correlation with riboflavin.
