# Supplementary material for: Evaluating Cefoperazone-Induced Gut Metabolic Functional Changes in MR1-Deficient Mice
Source: Metabolites. 2022 Apr 22;12(5):380. doi: 10.3390/metabo12050380 (PMC9146321; doi:10.3390/metabo12050380)
Supplement: Supplementary file 1 [file metabolites-12-00380-s001.zip › metabolites-1633073-supplementary.pdf]

For the best experience, open this PDF portfolio in Acrobat X or Adobe Reader X, or later.

Get Adobe Reader Now!
